# Supplementary material for: Genome-Wide Identification and Functional Characterization of CesA10 and CesA11 Genes Involved in Cellulose Biosynthesis in Sugarcane
Source: Int J Mol Sci. 2025 Nov 14;26(22):11046. doi: 10.3390/ijms262211046 (PMC12652704; doi:10.3390/ijms262211046)
Supplement: Supplementary file 1 [file ijms-26-11046-s001.zip › ijms-3912066-supplementary.pdf]

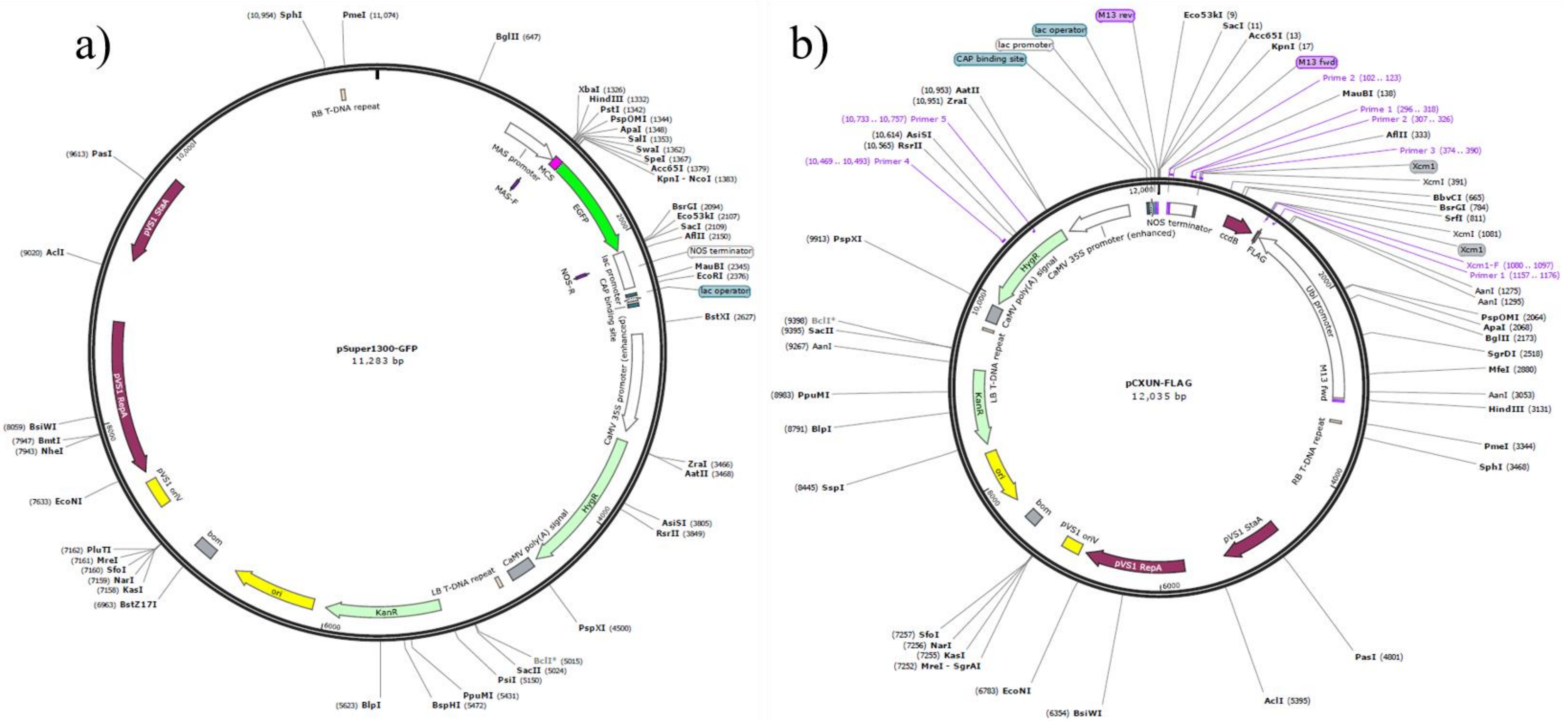

Supplementary Figure S1. Plasmid profile. a. pSuper1300. b. pCXUN FLAG.

Supplemental Table S1. The information of cellulose synthase gene in seven species

Gene name	Gene ID	Gene name	Gene ID	Gene name	Gene ID
AtCesA1	AT4G32410	OsCesA5	LOC_Os03g62090	ZmCesA9	GRMZM2G018241
AtCesA2	AT4G39350	OsCesA6	LOC_Os07g14850	ZmCesA10	GRMZM2G445905
AtCesA3	AT5G05170	OsCesA7	LOC_Os10g32980	ZmCesA11	GRMZM2G037413
AtCesA4	AT5G44030	OsCesA8	LOC_Os07g10770	ZmCesA12	GRMZM2G142898
AtCesA5	AT5G09870	OsCesA9	LOC_Os09g25490	VvCesA1	GSVIVG01035830001
AtCesA6	AT5G64740	OsCesA10	LOC_Os12g29300	VvCesA2	GSVIVG01023837001
AtCesA7	AT5G17420	OsCesA11	LOC_Os06g39970	VvCesA3	GSVIVG01032096001
AtCesA8	AT4G18780	SoCesA 1	Soffic.09G0009150	VvCesA4	GSVIVG01028402001
AtCesA9	AT2G21770	SoCesA 2	Soffic.10G0017310	VvCesA5	GSVIVG01013471001
AtCesA10	AT2G25540	SoCesA 3	Soffic.03G0004580	VvCesA6	GSVIVG01034552001
SbCesA1	Sb09g005280	SoCesA 5	Soffic.01G0017790	VvCesA7	GSVIVG01023643001
SbCesA2	Sb10g023430	SoCesA 7	Soffic.02G0008760	VvCesA8	GSVIVG01021248001
SbCesA3	Sb03g004320	SoCesA 8	Soffic.02G0007110	VvCesA9	GSVIVG01022285001
SbCesA5	Sb01g004210	SoCesA 9	Soffic.02G0032990	VvCesA10	GSVIVG01033297001
SbCesA6	Sb01g002050	SoCesA 10	Soffic.01G0033860	VvCesA11	GSVIVG01028234001
SbCesA7	Sb02g010110	SoCesA 11	Soffic.02G0033140	HvCesA1	HORVU.MOREX.r3.2HG0111610
SbCesA8	Sb02g007810	SoCesA 12	Soffic.02G0014650	HvCesA2	HORVU.MOREX.r3.5HG0530450
SbCesA9	Sb02g006290	ZmCesA1	GRMZM2G112336	HvCesA3	HORVU.MOREX.r3.5HG0522880
SbCesA10	Sb01g019720	ZmCesA2	GRMZM2G027723	HvCesA4	HORVU.MOREX.r3.1HG0041280
SbCesA11	Sb03g034680	ZmCesA3	GRMZM2G039454	HvCesA5	HORVU.MOREX.r3.3HG0288960
SbCesA12	Sb02g025020	ZmCesA4	GRMZM2G424832	HvCesA6	HORVU.MOREX.r3.6HG0550800
OsCesA1	LOC_Os05g08370	ZmCesA5	GRMZM2G111642	HvCesA7	HORVU.MOREX.r3.1HG0027850
OsCesA2	LOC_Os03g59340	ZmCesA6	GRMZM2G113137	HvCesA8	HORVU.MOREX.r3.5HG0482670
OsCesA3	LOC_Os07g24190	ZmCesA7	GRMZM2G025231	HvCesA9	HORVU.MOREX.r3.2HG0115540
OsCesA4	LOC_Os01g54620	ZmCesA8	GRMZM2G177631	HvCesA10	HORVU.MOREX.r3.7HG0638850

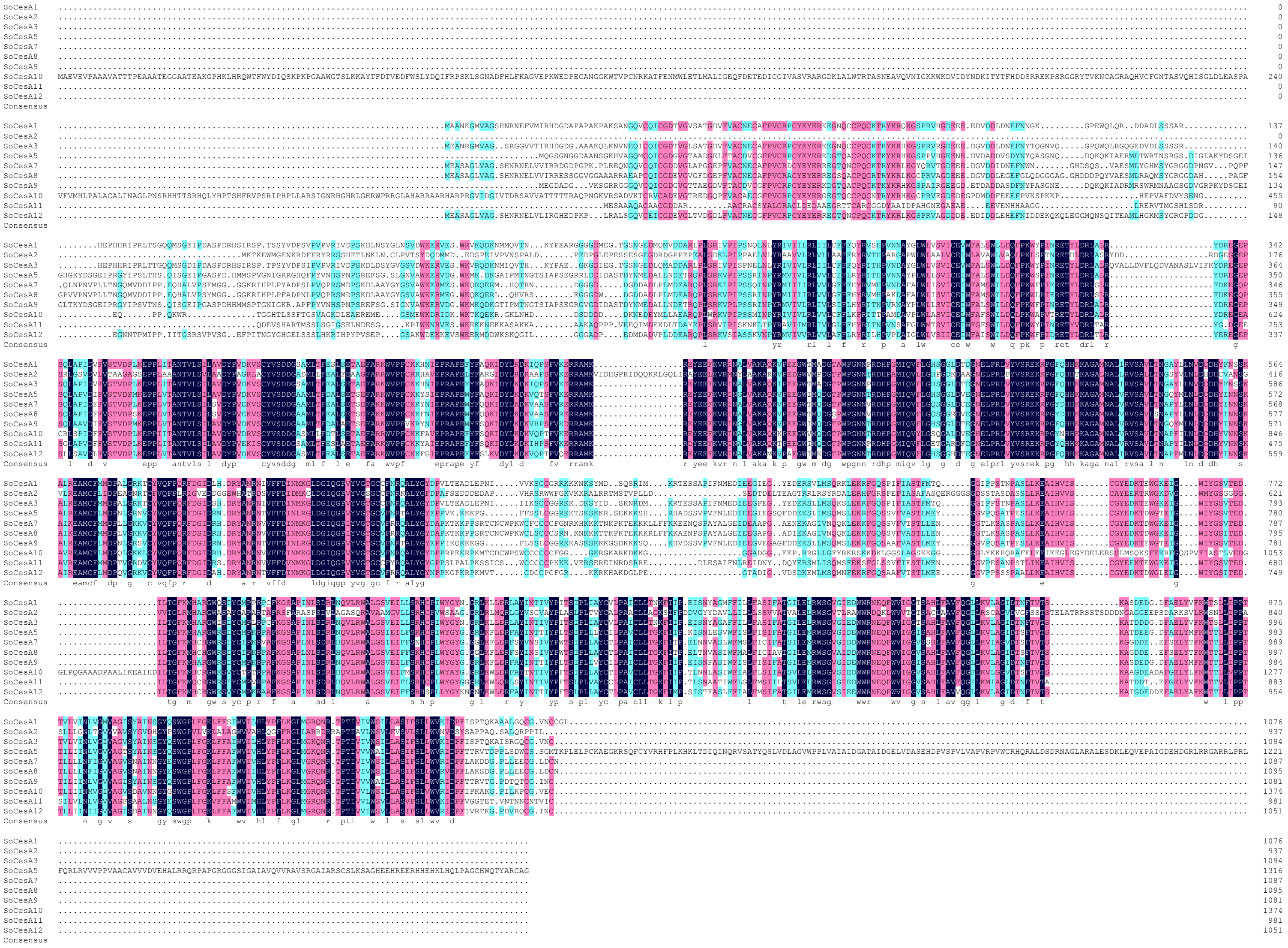

Supplementary Figure S2. CESA protein sequence alignment diagram.

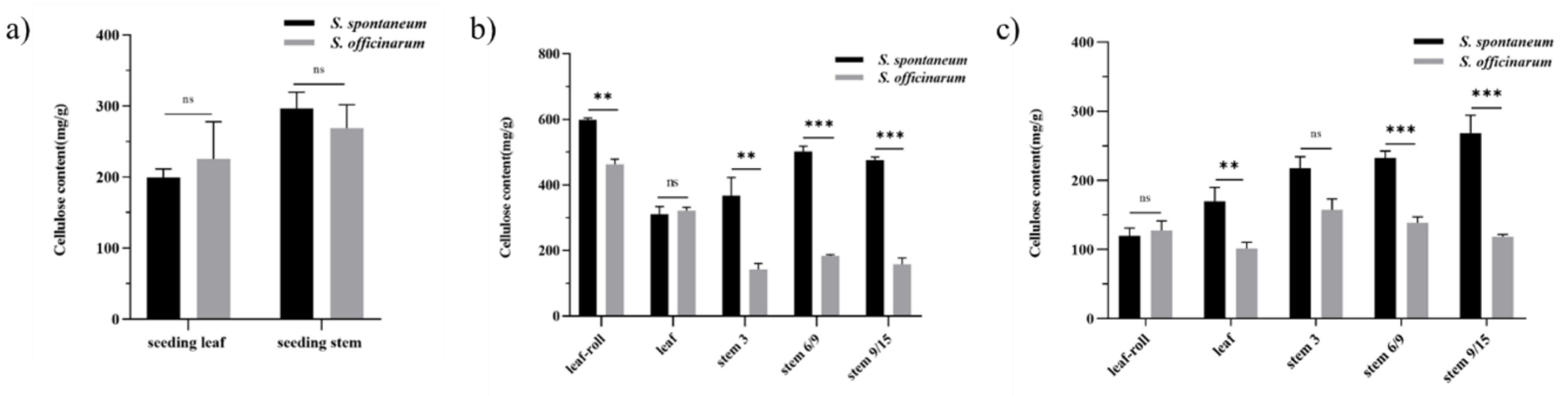

Supplementary Figure S3. Cellulose content of two *Saccharum* species in different tissues and different developmental stages. a-c: Represent the results of cellulose content measurements in different tissues of the *S. spontaneum* and *S. officinarum* during the seedling stage, pre-mature stage, and mature stage. Error bars, SD (n = 3). * indicates significant differences at $p < 0.05$.

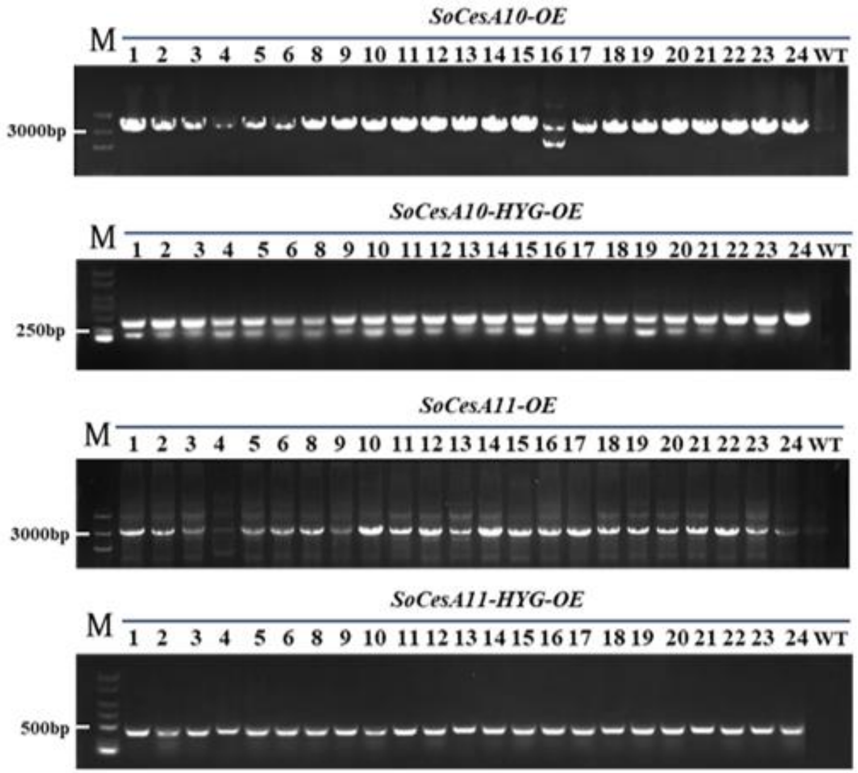

Supplementary Figure S4. Identification of overexpression-positive seedlings.
